# Bcl-xL promotes metastasis independent of its anti-apoptotic activity

**DOI:** 10.1038/ncomms10384

**Published:** 2016-01-20

**Authors:** Soyoung Choi, Zhengming Chen, Laura H. Tang, Yuanzhang Fang, Sandra J. Shin, Nicole C. Panarelli, Yao-Tseng Chen, Yi Li, Xuejun Jiang, Yi-Chieh Nancy Du

**Affiliations:** 1 Department of Pathology and Laboratory Medicine, Weill Cornell Medicine, New York, New York 10065, USA; 2 Division of Biostatistics and Epidemiology, Department of Healthcare Policy and Research, Weill Cornell Medicine, New York, New York 10065, USA; 3 Department of Pathology, Memorial Sloan Kettering Cancer Center, New York, New York 10065, USA; 4 Lester and Sue Smith Breast Center, Baylor College of Medicine, Houston, Texas 77030, USA; 5 Cell Biology Program, Memorial Sloan Kettering Cancer Center, New York, New York 10065, USA

## Abstract

Bcl-xL suppresses mitochondria-mediated apoptosis and is frequently overexpressed in cancer to promote cancer cell survival. Bcl-xL also promotes metastasis. However, it is unclear whether this metastatic function is dependent on its anti-apoptotic activity in the mitochondria. Here we demonstrate that Bcl-xL promotes metastasis independent of its anti-apoptotic activity. We show that apoptosis-defective Bcl-xL mutants and an engineered Bcl-xL targeted to the nucleus promote epithelial–mesenchymal transition, migration, invasion and stemness in pancreatic neuroendocrine tumour (panNET) and breast cancer cell lines. However, Bcl-xL proteins targeted to the mitochondria or outside of the nucleus do not have these functions. We confirm our findings in spontaneous and xenograft mouse models. Furthermore, Bcl-xL exerts metastatic function through epigenetic modification of the TGFβ promoter to increase TGFβ signalling. Consistent with these findings, we detect nuclear Bcl-xL in human metastatic panNETs. Taken together, the metastatic function of Bcl-xL is independent of its anti-apoptotic activity and its residence in the mitochondria.

## Abstract (web summary)

Bcl-xL is an anti-apoptotic protein that has also been implicated in metastasis. In this study, the authors show that nuclear Bcl-xL promotes metastasis by regulating TGFβ signaling, which is independent of the anti-apoptotic activity of Bcl-xL.

## Graphical abstract

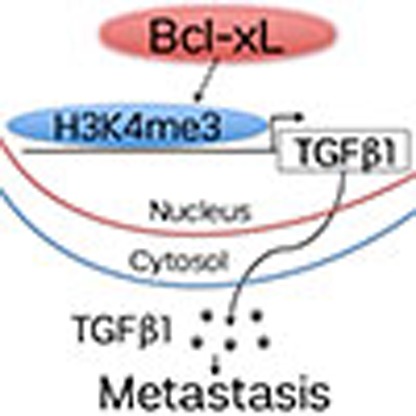

Cancer cells evade apoptosis through upregulation of anti-apoptotic proteins and/or downregulation of pro-apoptotic proteins^1^,^2^. The Bcl-2 family members are the key regulators of apoptosis and can be subdivided into anti-apoptotic members and pro-apoptotic members^3^. Anti-apoptotic Bcl-2 family members are overexpressed in a variety of cancers through genetic alterations, such as chromosomal translocation (Bcl-2) or amplification (Bcl-xL and Mcl-1)^4^,^5^,^6^. These anti-apoptotic proteins contain a hydrophobic groove that binds to the pro-apoptotic proteins, Bax and Bak, which are essential effectors responsible for mitochondrial outer membrane (MOM) permeabilization. The balance between these two opposing members is critical in determining the cell fate. In healthy cells, Bax and Bak generally are held in check by the anti-apoptotic Bcl-2 proteins. In response to apoptotic stimuli, the third Bcl-2 subfamily, BH3-only proteins, promote apoptosis by either activating Bax and Bak or inactivating Bcl-2, Bcl-xL and Mcl-1 (ref. 7). Subsequently, Bax and Bak are recruited to the MOM, where they oligomerize and cause MOM permeabilization, releasing pro-apoptotic effectors such as cytochrome c and SMAC (the second mitochondria-derived activator of caspase). The released pro-apoptotic factors then activate caspases and a series of downstream events, ultimately resulting in cell death^8^. Overexpression of anti-apoptotic Bcl-2 proteins in cancers tilts the balance towards cell survival. Pharmacological inhibition of anti-apoptotic Bcl-2 proteins in cancer has emerged as a major strategy to induce apoptosis and tumour regression^9^.

New evidence from our studies and others suggests that, in addition to the regulation of apoptosis, Bcl-2 members may possess other biological functions^10^,^11^. Using a mouse model of spontaneous multistep tumorigenesis, *RIP-Tag; RIP-tva*, we have previously identified a novel function of Bcl-xL in promoting invasion and metastasis of pancreatic neuroendocrine tumour (panNET)^12^. Surprisingly, we did not detect significant differences in apoptotic rates either in the tumours from mice infected with Bcl-xL or in mouse panNET cells (N134) overexpressing Bcl-xL *in vitro*^12^. Furthermore, we demonstrated that Bcl-xL remodelled cell morphology and induced epithelial–mesenchymal transition (EMT) in a mouse panNET cell line (N134)^12^. Our results are consistent with a subsequent study showing that the incidence of invasive islet tumours was reduced in mice harbouring pancreatic β-cell-specific knockout of Bcl-x (for both Bcl-xL and Bcl-xS), and that the tumour cells lacking Bcl-x were impaired in invasion *in vitro* under conditions mimicking hypoxia^13^. In these Bcl-x null tumours, the expression levels of other anti-apoptotic Bcl-2 family members were not significantly altered, suggesting that there was no compensatory transcriptional upregulation^13^. Besides in panNET, knockdown of Bcl-xL impairs migration of colorectal cancer cell lines *in vitro*, while overexpression promotes their migration^14^. Overexpression of Bcl-xL also induces EMT in lung cancer cell lines *in vitro*, and it increases invasiveness of glioma cell lines and metastasis of breast cancer cell lines in xenograft models^15^,^16^,^17^,^18^,^19^,^20^. Overexpression of Bcl-xL in breast cancer has been shown to be associated with high tumour grade, local invasion into stroma and nodal metastases^21^,^22^.

However, it remains to be determined whether this novel metastatic function of Bcl-xL is the consequence of the well-known anti-apoptotic function of Bcl-xL or of another activity that is separate from its roles in inhibiting apoptosis. In this report, by performing a series of cellular and mouse model experiments, we demonstrate that Bcl-xL promotes EMT, cell migration and metastasis, independent of its anti-apoptotic activity and that nuclear Bcl-xL, but not mitochondrial Bcl-xL or Bcl-xL outside of the nucleus, is responsible for this function.

## Results

### Bcl-xL promotes migration DKO MEFs *in vitro* and *in vivo*

**Figure 1:**
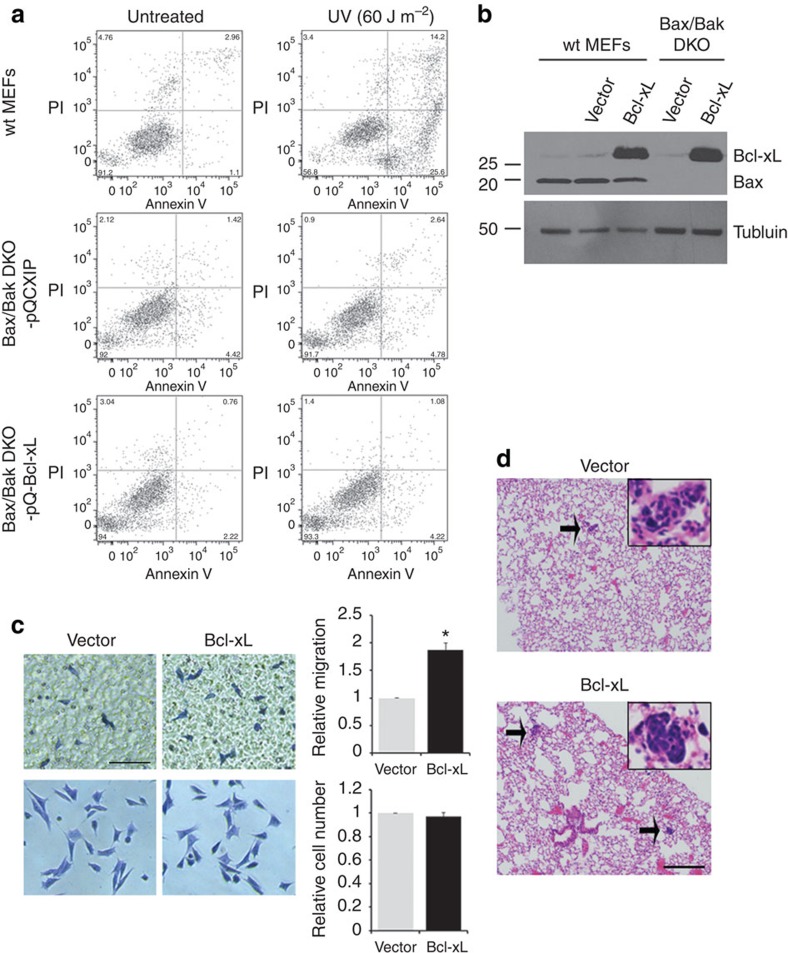
Bcl-xL promotes migration in the absence of pro-apoptotic proteins Bax/Bak in MEFs. (**a**) Wild-type (wt) MEFs and Bax/Bak DKO MEFs overexpressing control vector (pQCXIP) or Bcl-xL were untreated or treated with 60 J m^−2^ UV. After 24 h, apoptosis was measured by staining cells with Annexin V and propidium iodide (PI). Only wt MEFs demonstrated the increased Annexin V-positive cells. (**b**) Western blot analysis for the level of Bcl-xL proteins in wt MEFs and Bax/Bak DKO MEFs overexpressing control vector (pQCXIP) or Bcl-xL. α-tubulin was used a loading control. (**c**) Migration of Bax/Bak DKO MEFs overexpressing control vector or Bcl-xL was determined using *in vitro* transwell migration chamber with a serum gradient (2–10%). Overall, 5 × 10^4^ cells were seeded in the upper chambers of the transwell inserts. Four hours later, cells attached on the top of the upper chambers were removed, and the number of cells on the bottom surface of the transwell inserts was counted. Bax/Bak DKO MEFs overexpressing Bcl-xL demonstrated enhanced migration compared with Bax/Bak DKO MEFs overexpressing control vector (top row), and the relative cell numbers between the two cell lines remained the same in a regular cell culture condition (bottom row). Following crystal violet staining, cells were counted from eight randomly picked fields in three independent experiments. Error bars represent s.e.m. *P=0.02 relative to control (vector), two-sided *t*-test. Scale bar, 100 μm. Original magnification, × 20. (**d**) Lung sections from mice injected with Bax/Bak DKO MEFs overexpressing control vector or Bcl-xL through lateral tail vein were stained with haematoxylin and eosin. Bax/Bak DKO MEFs overexpressing Bcl-xL developed more micrometastatic foci (arrows) compared with control Bax/Bak DKO MEFs. Scale bar, 200 μm. Original magnification, × 40 for inserted photos.

To examine whether the canonical anti-apoptotic function is required for Bcl-xL-mediated cell migration and metastasis, we first used Bax/Bak double knockout (DKO) mouse embryonic fibroblasts (MEFs), which are defective in mitochondria-mediated apoptosis. We verified that the Bax/Bak DKO cell line was resistant to apoptosis following ultraviolet (UV) treatment of radiation, while the control wild-type MEFs became apoptotic following the same treatment (Fig. 1a).

To assess whether Bcl-xL promotes migration in the absence of Bax/Bak, we performed an *in vitro* transwell migration assay. We seeded Bax/Bak DKO cells overexpressing the control vector or Bcl-xL (Fig. 1b) on the upper chambers of transwell inserts with 8-μm porous polycarbonate membranes. We then measured cell migration along a serum gradient through the membrane after 4 h of incubation. We found that, although Bcl-xL did not protect these Bax/Bak DKO cells from UV-induced apoptosis, Bcl-xL was able to promote migration in the absence of Bax and Bak (Fig. 1a,c). To ensure that any increase in cell migration was not due to an increase in cell proliferation, we measured cell proliferation of Bax/Bak DKO cells overexpressing the control vector or Bcl-xL. Indeed, there was no significant difference in cell proliferation between these two cell lines during the 4 h of incubation (Fig. 1c). Of note, the above findings were confirmed using another independent clone, demonstrating that the effect of Bcl-xL in promoting cell migration is not a caveat of plasmid insertion deregulating endogenous genes crucial in cell migration (Supplementary Fig. 1).

To investigate whether Bcl-xL promotes metastasis of Bax/Bak DKO cells *in vivo*, we performed an experimental tail vein metastasis assay. We injected 0.5 × 10^5^ Bax/Bak DKO cells overexpressing control vector or Bcl-xL into the tail vein of NOD/scid-lL2Rgc knockout (NSG) immunodeficient mice. Four weeks later, we analysed the capacity of these cells in forming lung metastases. Overall, 43% of mice receiving DKO cells overexpressing the control vector developed lung tumour nodules, while 75% of mice receiving DKO cells overexpressing Bcl-xL developed lung tumour nodules (Fig. 1d and Table 1). Histological analysis of the lung section revealed that mice receiving DKO cells overexpressing the control vector had 2.3±1.2 metastatic foci, while mice receiving DKO cells overexpressing Bcl-xL had 16.3±9.4 metastatic foci (Table 1, *P*=0.0046, Wilcoxon rank-sum test), suggesting that Bcl-xL increases the capacity in forming lung metastases in a Bax/Bak-independent manner.

### Bcl-xL mutants promote migration of MEFs

**Figure 2:**
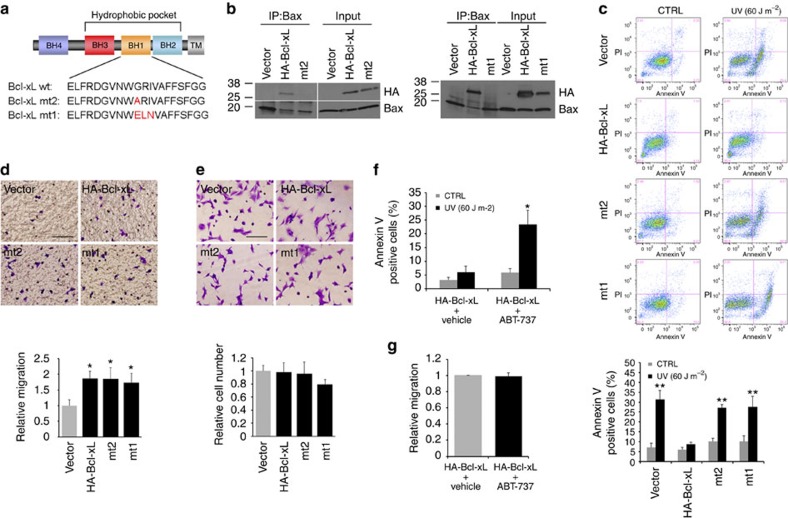
Bcl-xL mutants that fail to bind to Bax/Bak promotes migration. (**a**) Schematic diagram of Bcl-xL constructs. BH, Bcl-2 homology domain; TM, transmembrane domain. (**b**) Cell lysates of wt MEFs overexpressing control vector, HA-Bcl-xL (wt), HA-Bcl-xL mt2 and HA-Bcl-xL mt1 were subjected to immunoprecipitation with Bax antibody and then followed by western blot analysis to detect HA-Bcl-xL proteins and Bax. Both Bcl-xL mutants (mt2 and mt1) lost the ability to bind Bax in MEFs. (**c**) wt MEFs overexpressing control vector, HA-Bcl-xL, HA-Bcl-xL-mt2 and HA-Bcl-xL-mt1 were treated with 60-J m^−2^ UV, and then apoptosis was examined using flow cytometry analysis following Annexin V and PI staining. Only HA-Bcl-xL (wt) prevented UV-induced apoptosis. **P<0.01 relative to vector CTRL. *N*=4 independent experiments, and error bar represents s.e.m. Data were analysed using ANOVA, followed by Dunnett's *post hoc* test. (**d**,**e**) wt MEFs overexpressing three Bcl-xL constructs (wt-Bcl-xL, mt2 and mt1) increased cell migration compared with wt MEFs overexpressing control vector (**d**). Migration of cells was determined using *in vitro* transwell migration chamber, as described in Fig. 1c. The relative cell numbers among the cell lines remained the same in a regular cell culture condition (**e**). Cells were counted from eight randomly picked fields in five independent experiments in both **d**,**e**. *P<0.04 relative to vector alone. Values are means±s.e.m. Data were analysed using ANOVA, followed by Dunnett's *post hoc* test. Scale bar, 100 μm. Original magnification, × 20. (**f**) wt MEFs overexpressing HA-Bcl-xL were pretreated with ABT-737 (10 μM) for 2 h before UV (60 J m^−2^) treatment. After 24 h, apoptosis was measured by staining cells with Annexin V and PI. ABT-737 reversed the anti-apoptotic effect of Bcl-xL. *P<0.05 relative to vehicle-treated HA-Bcl-xL. *N*=4 independent experiments, and error bar represents s.e.m. Data were analysed using ANOVA, followed by Dunnett's *post hoc* test. (**g**) wt MEFs overexpressing HA-Bcl-xL pretreated with 10 μM ABT-737 did not affect the Bcl-xL-mediated cell migration. Migration of cells was determined using *in vitro* transwell migration chamber, as described in Fig. 1c.

To further confirm that the metastatic function of Bcl-xL is independent of its anti-apoptotic function, we utilized two well-established Bcl-xL mutants that are defective in the anti-apoptotic function. Bcl-xL mutant 1 (mt1) has a GRI (residues 138–140) to ELN substitution in the BH1 domain, and mutant 2 (mt2) has a G (residue 138) to A substitution in the BH1 domain (Fig. 2a). These mutants are impaired in binding to Bax and cannot protect cells from apoptosis^23^,^24^. Only wild-type Bcl-xL, but not the two mutants, co-immunoprecipitated with Bax and other pro-apoptotic Bcl-2 family members (Fig. 2b and Supplementary Fig. 2). Only wild-type Bcl-xL, but not the two mutants, protected MEFs from apoptosis induced by UV (Fig. 2c) or from anoikis induced by cell detachment (Supplementary Fig. 3). To ascertain whether the two Bcl-xL mutants promote cell migration, we performed an *in vitro* transwell migration assay. Both mutants as well as wild-type Bcl-xL promote cell migration (Fig. 2d). There was no significant difference in cell proliferation when comparing MEFs overexpressing the Bcl-xL mutants with MEFs overexpressing the control vector (Fig. 2e).

In addition to using the Bcl-xL mutants defective in anti-apoptotic function, we took a pharmacological approach to inhibit the anti-apoptotic function of Bcl-xL. ABT-737, a Bcl-2 homology domain 3 (BH3) mimetic, was designed to block the ability of Bcl-xL to bind to Bax/Bak^5^. To examine whether ABT-737 affects the ability of Bcl-xL-mediated cell migration, MEFs overexpressing wild-type Bcl-xL were pretreated with ABT-737 (10 μM) and then subjected to an *in vitro* transwell migration assay. Even though ABT-737 inhibited the anti-apoptotic effect of Bcl-xL (Fig. 2f), it did not reduce Bcl-xL-mediated migration (Fig. 2g). Collectively, these data suggest that Bcl-xL can promote cell migration independent of its anti-apoptosis activity.

### Bcl-xL promotes lymph node metastasis in a model of panNETs

**Figure 3:**
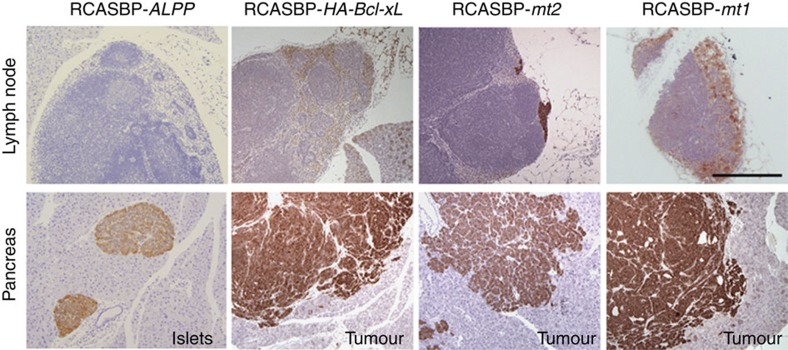
HA-Bcl-xL mutants defective in anti-apoptotic function promote metastasis of primary panNETs in *RIP-Tag; RIP-tva* mice. *RIP-Tag; RIP-tva* mice were infected with the indicated RCASBP retroviruses at 7 weeks of age and killed at 16 weeks of age. Organs were harvested for histological analysis. While mice with RCASBP-*ALPP* did not develop lymph node metastasis, lymph node metastasis was observed in mice with RCASBP-*HA-Bcl-xL*, *mt2* and *mt1*. Photographs show representative synaptophysin staining of metastatic panNET in pancreatic lymph nodes (upper panel) and islets or tumours in pancreatic sections (lower panel) of *RIP-Tag; RIP-tva* mice infected with RCASBP-*ALPP*, RCASBP-*HA-Bcl-xL*, *mt2* and *mt1*. Scale bar, 200 μm.

We have previously shown that Bcl-xL promotes lymph node metastasis of primary panNETs in the *RIP-Tag; RIP-tva* mouse model, which faithfully models multiple steps of tumour development in human cancer and allows for genetic alterations to be introduced into premalignant lesions by infection with avian retroviral vector, RCASBP^12^. To evaluate whether the Bcl-xL mutants defective in anti-apoptotic activity retain the capacity to promote lymph node metastasis of panNETs *in vivo*, we employed the *RIP-Tag; RIP-tva* mouse model. We generated RCASBP viruses encoding the cDNA for the two haemagglutinin (HA)-tagged Bcl-xL mutants defective in anti-apoptotic function (mt 1 and mt 2) and HA-tagged wild-type Bcl-xL. We injected high-titre virus stocks (0.1 ml; >10^8^ infectious units per millilitre) into 7-week-old *RIP-Tag; RIP-tva* mice intracardially. RCASBP-*ALPP* (alkaline phosphatase)^25^ was used as a negative control for adverse effects of viral infection *per se*. Nine weeks after infection, we harvested the pancreas and other organs for histological staging and grading of the lesions. To aid the search for metastasis of panNET in *RIP-Tag; RIP-tva* mice, tissue sections were subjected to immunohistochemical staining for synaptophysin, a neuroendocrine marker. Pancreatic lymph node metastases were readily observed in mice infected with RCASBP-*HA-Bcl-xL*, as predicated based on our previous work using untagged Bcl-xL^12^, which demonstrates that an N-terminal HA-epitope tag does not interfere with the metastatic function of Bcl-xL. In mice infected with either RCASBP-*HA-Bcl-xL mutant 1* or *mutant 2*, pancreatic lymph node metastases were also found at 16 weeks of age and with a similar frequency (4/6 and 5/9 mice, respectively) compared to mice injected with virus carrying wild-type Bcl-xL (4/7 mice; Fig. 3). RCASBP-*ALPP* control-injected mice did not form lymph node metastases (0/8 mice). These data suggest that the ability of Bcl-xL mutants defective in anti-apoptotic function in promoting lymph node metastasis of panNET is comparable to that of wild-type Bcl-xL in this *RIP-Tag; RIP-tva* mouse model.

### Bcl-xL mutants promote EMT and migration of panNET cells

**Figure 4:**
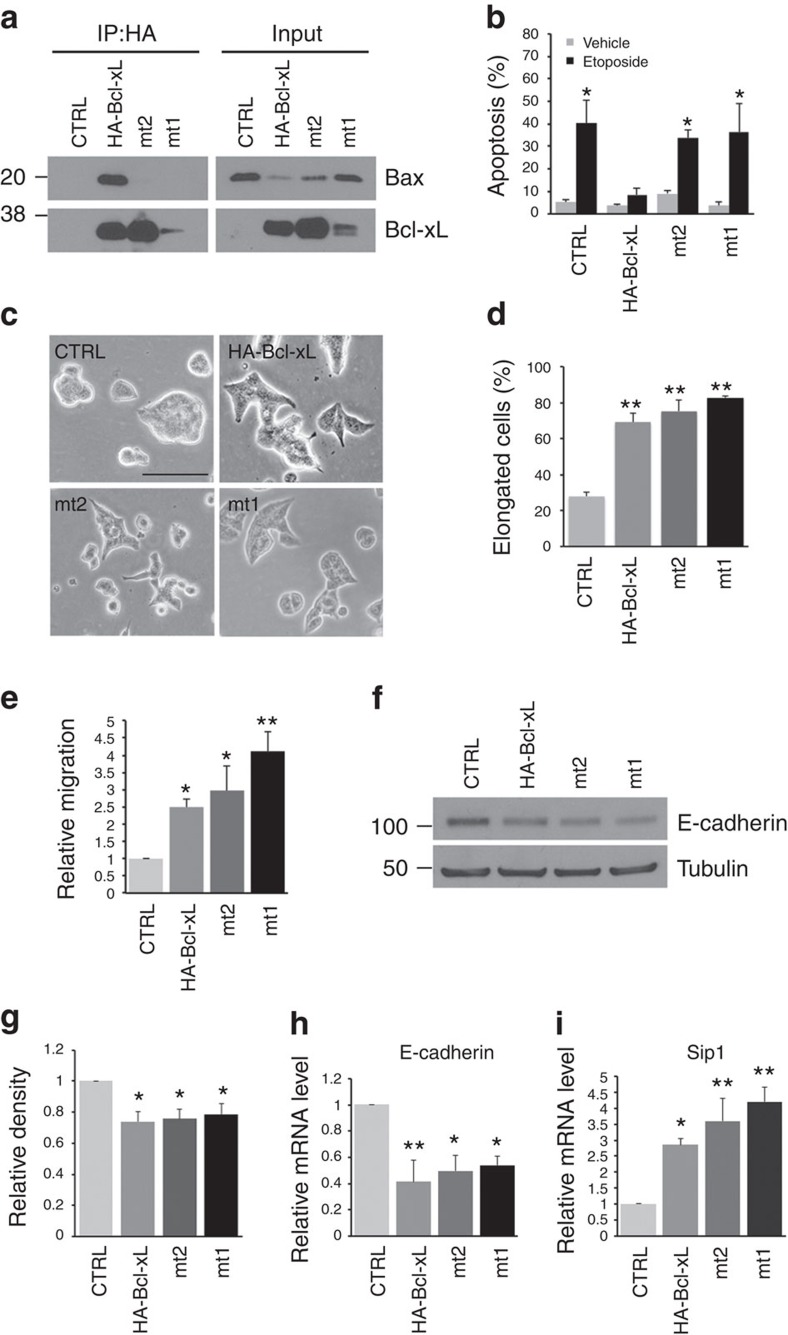
Bcl-xL mutants defective in anti-apoptotic function retain the effect of wt Bcl-xL on cell migration and EMT in the mouse N134 panNET cell line. (**a**) Cell lysates were subjected to immunoprecipitation with HA magnetic beads and then followed by western blot analysis to detect Bcl-xL and Bax. Both Bcl-xL mutants (mt2 and mt1) lost the ability to bind to Bax in N134. (**b**) N134 tumour cells infected with RCASBP-*HA-β-actin*, *HA-Bcl-xL*, *mt2* and *mt1* were treated with 10-μM etoposide for 24 h, and then apoptosis was examined with flow cytometry analysis following Annexin V and PI staining. Only cells infected with HA-Bcl-xL prevented etoposide-induced apoptosis. *P<0.05 relative to CTLR treated with vehicle. Values are means±s.e.m., *N*=4. Data were analysed using ANOVA from four independent experiments, followed by Dunnett's *post hoc* test. (**c**) Bright-field images of N134 tumour cells. Cells infected with RCASBP-*HA-Bcl-xL*, *mt2* or *mt1* changed morphology compared with control cells (RCASBP-*HA-β-actin*). Scale bar, 50 μm. (**d**) Quantification of the proportion of elongated cells shown in **c**. Values are means±s.e.m., *N*=4. **P<0.01 compared with CTRL, one-way ANOVA with Dunnett's *post hoc* test. (**e**) Migration of N134 tumour cells was determined using *in vitro* transwell migration chamber. Data are expressed as the normalized number of migrated cells in the bottom chambers in eight fields under × 20 magnification after 72 h relative to that of cells infected with RCASBP-*HA-β-actin* (CTRL) from three independent experiments. Values are means±s.e.m., *N*=3. *P<0.05, **P<0.01 compared with CTRL, one-way ANOVA with Dunnett's *post hoc* test. (**f**) Western blot analysis for E-cadherin and α-tubulin (as a loading control). E-cadherin was reduced in N134 tumour cells infected with RCASBP-*HA-Bcl-xL*, *mt2* and *mt1* compared with cells infected with RCASBP-*HA-β-actin*. Data shown are representative of six independent experiments. (**g**) Densitometric values were normalized to tubulin and are expressed relative to the CTRL from six blots. *P< 0.05 compared with CTRL, one-way ANOVA with Dunnett's *post hoc* test. (**h**,**i**) Quantitative real-time PCR analysis of E-cadherin and Sip1 mRNA in N134 tumour cells. *P<0.05, **P<0.01. *N*=4 independent experiments, and error bar represents s.e.m. Data was analysed using ANOVA, followed by Dunnett's *post hoc* test.

We previously demonstrated that overexpression of Bcl-xL induces EMT by changing cell morphology, increasing cell motility and reducing protein levels of E-cadherin in a mouse panNET cell line, N134 (ref. 12). To investigate whether the two Bcl-xL mutants can also promote EMT of N134, we infected N134 with RCASBP-*mt1*, RCASBP-*mt2* or RCASBP-*HA-Bcl-xL in vitro*. To verify the expression of these different HA-tagged Bcl-xL proteins and the loss of binding affinity to Bax, we performed western blot analysis and immunoprecipitation experiments with anti-HA magnetic beads. The immunoprecipitates were subjected to western blot analysis using antibodies against HA or Bax. We confirmed that only wild-type HA-Bcl-xL, but not the two mutants, co-immunoprecipitated with Bax in N134 cells (Fig. 4a). We also verified that the two mutants could not protect N134 cells from apoptosis induced by etoposide (topoisomerase II inhibitor; Fig. 4b). The N134 tumour cells overexpressing either Bcl-xL mutants or wild-type HA-Bcl-xL became more elongated as compared to the control N134 tumour cells infected with RCASBP-*HA-β-actin*^26^ ∼3 weeks after infection with RCASBP viruses (Fig. 4c,d). As expected, these cells overexpressing wild-type or mutant Bcl-xL migrated more than control tumour cells in response to a serum gradient in the transwell migration assay (Fig. 4e). Cell migration is a characteristic feature of EMT and metastasis. These results suggest that the Bcl-xL mutants defective in anti-apoptotic function retain the ability to change cell morphology and to promote tumour cell migration.

In addition to morphological changes indicative of EMT, we determined whether Bcl-xL mutants defective in anti-apoptotic function also induced molecular markers of EMT. Both Bcl-xL mutants downregulated E-cadherin in both protein and mRNA levels (Fig. 4f–h). These mutants also significantly increased the mRNA level of Smad interacting protein 1 (sip1), an EMT transcription factor (Fig. 4i). Taken together, these results reveal that the capacity of Bcl-xL in regulating cell morphology, EMT and migration in mouse panNET cells is independent of its anti-apoptotic function.

### Bcl-xL mutants enhance the metastasis of human panNET cells

**Figure 5:**
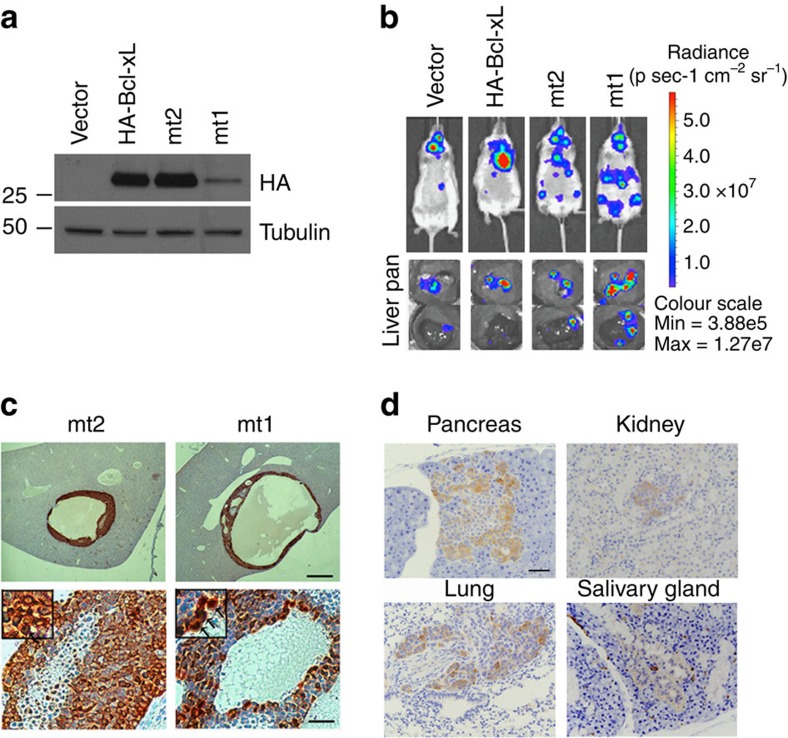
Bcl-xL mutants defective in anti-apoptotic function enhance metastasis of BON1-TGL cells *in vivo.* (**a**) The expression of HA-Bcl-xL, mt2 or mt1 in BON1-TGL infected with the retroviruses of pQCXIP vector bearing each construct was confirmed with western blot analysis for HA and α-tubulin (as a loading control). (**b**) Representative bioluminescent images of NSG mice (upper panel) and their pancreases and livers (lower panel). Intracardiac injections of BON1-TGL cells overexpressing mt2 and mt1 developed highly aggressive metastasis throughout the body. A total of 1 × 10^6^ cells were injected into the left ventricle of five recipient NSG mice for each group. The location of BON1-TGL cells was monitored by *in vivo* bioluminescent imaging 4 weeks after injection. Bioluminescent signal was detected in several organs including the pancreas and liver right after the mice were killed. (**c**) Immunohistochemical staining of HA in the liver sections from NSG mice injected with BON1-TGL overexpressing mt2 and mt1. Lower panel shows high magnification of immunostained liver sections. Note the nuclear staining of HA-mt1 (arrows) in the inserted photo. Scale bar, 500 μm for upper panel and 50 μm for lower panel. Original magnification × 40 for inserted photos. (**d**) Representative metastases found in the multiple organs of mice injected with BON1-TGL cells overexpressing mt1. Immunohistochemical staining of luciferase on the sections of pancreas, kidney, lung and salivary gland was shown. Scale bar, 50 μm. Original magnification, × 40.

In our previous experience, mouse panNET cells are poorly metastatic in experimental metastasis assay^27^, and may not be adequate for comparing the metastasis-promoting capacity of our Bcl-xL mutants. Therefore, to further determine whether the two Bcl-xL mutants as well as wild-type Bcl-xL can enhance metastasis of panNET cells, we employed the human panNET cell line, BON1, which is the most commonly utilized human panNET cell line and was established from a peri-pancreatic lymph node in a patient with metastatic panNET^28^. To detect the localization and growth of the tumour cells inside mice using *in vivo* luciferase bioluminescence imaging, we infected the BON1 cell line with viruses carrying thymidine kinase/green fluorescent protein/luciferase fusion reporter (TGL)^29^ and sorted for green fluorescent protein (GFP)-positive cells (BON1-TGL). We generated BON1-TGL cells overexpressing the control vector, wild-type Bcl-xL or the two mutants (mutants 1 and 2). The expression levels of wild-type Bcl-xL and mutant 2 were similar, while Bcl-xL mutant 1 had the lowest level of Bcl-xL proteins (Fig. 5a). We injected a total of 1 × 10^6^ cells into NSG immunodeficient mice with intracardiac injection. Mice were monitored by *in vivo* luciferase bioluminescence imaging for 4 weeks. Wild-type Bcl-xL enhanced metastasis of BON1-TGL in mice, primarily in the lungs. BON1-TGL cells overexpressing mutants 1 and 2 developed extensive metastases throughout the body (Fig. 5b, upper panels). After the mice were killed, the major organs were immediately harvested for luciferase bioluminescence imaging *ex vivo* and further histological analysis. As shown in Fig. 5b (bottom panels), pancreatic and liver signals were intense from mice receiving BON1-TGL cells overexpressing mutants 1 and 2. We also confirmed that the metastatic tumours indeed expressed HA-Bcl-xL (Fig. 5c). Importantly, a significant proportion of the Bcl-xL proteins was found in the nucleus of these metastatic tumour cells (Fig. 5c). In addition, immunohistochemical staining using antibodies against luciferase or HA also detected metastatic BON1-TGL cells in the pancreas, kidney, lung and salivary gland (Fig. 5d). Together, these data suggest that Bcl-xL mutants defective in the anti-apoptotic function promote metastasis of human panNET cells *in vivo*.

### Bcl-xL overexpression increases TGFβ levels

**Figure 6:**
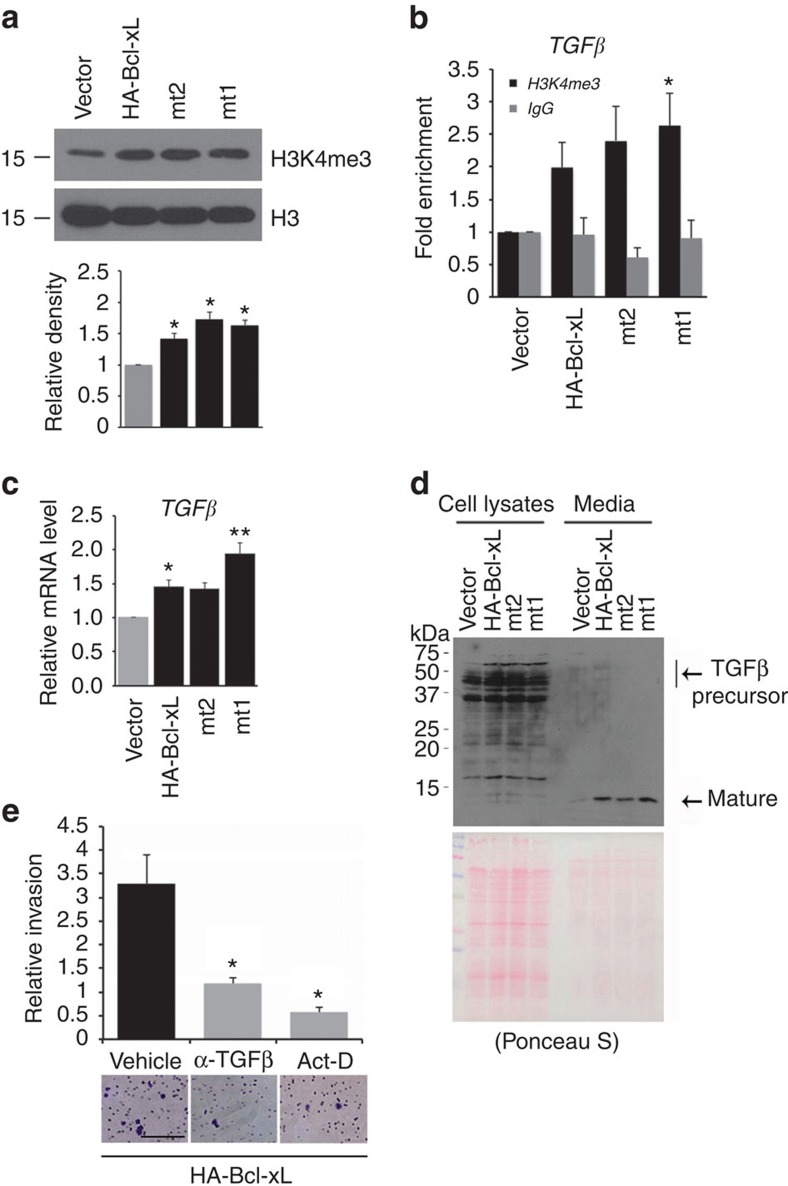
Bcl-xL overexpression in BON1-TGL cells increases TGFβ levels in a Bax/Bak-independent manner. (**a**) Western blot analysis for H3K4me3 and total H3 (as a loading control). H3K4me3 was increased in BON1-TGL cells overexpressing HA-Bcl-xL, mt2 and mt1 compared with vector alone. Bar graph showing densitometric values normalized to H3 and expressed relative to the control from three blots. *P<0.05 compared with the control, one-way ANOVA with Holm's *post hoc* test. (**b**) Real-time quantitative PCR analysis of immunoprecipitates from ChIP using anti-H3K4me3 antibodies or normal rabbit serum for enrichment of *TGFβ* promoter. Data were calculated as fold enrichment over the input control with error bars (means±s.e.m.) from three independent experiments. *P<0.05, compared with control, one-way ANOVA with Tukey's *post hoc* test. (**c**) *TGFβ* mRNA was increased in BON1-TGL cells overexpressing HA-Bcl-xL, mt2 and mt1 compared with BON1-TGL cells overexpressing vector alone from three independent experiments. Values are means±s.e.m., *N*=3. *P<0.05, **P<0.01 compared with vector alone, one-way ANOVA with Dunnett's *post hoc* test. (**d**) Western blot shows TGFβ precursor and mature TGFβ in cell lysates and media from BON1-TGL cells overexpressing HA-Bcl-xL, mt2 or mt1 compared with BON1-TGL cells overexpressing vector alone. Mature TGFβ was strongly detected in culture medium from BON1-TGL cells with HA-Bcl-xL, mt2 and mt1. This blot is representative of three independent experiments. Blots were stained with Ponceau S to ensure equal protein loading and transfer. Data shown are representative of three independent experiments. (**e**) Invasion of BON1-TGL cells overexpressing HA-Bcl-xL was determined using *in vitro* matrigel-coated transwell invasion chamber. Cells were pretreated with α-TGFβ (2 μg ml^−1^) or Actinomycin D (0.01 μg ml^−1^) for 2 h and 2 × 10^4^ cells were plated in the upper chambers of transwell inserts in the presence of the respective drugs. Data are expressed as the normalized number of cells migrated to the bottom surface of the transwell inserts in eight fields under × 20 magnification after 24 h relative to that of cells with vector alone. Values are means±s.e.m., *N*=3. *P<0.05, compared with vehicle-treated HA-Bcl-xL, one-way ANOVA with Holm's *post hoc* test. Scale bar, 100 μm.

Because a time delay appears to be needed for the transfected Bcl-xL, mt2 or mt1 to change cell morphology (Fig. 4c), we tested whether Bcl-xL exerts its metastatic functions via epigenetic reprogramming and transcriptional changes. We found that histone H3 trimethyl Lys4 (H3K4me3), an active mark for transcription, was upregulated in cells overexpressing Bcl-xL, mutant 2 and mutant 1 compared with that in control cells (Fig. 6a).

Transforming growth factor β (TGFβ) gene transcription is known to be regulated by epigenetic reprogramming^30^, and has been previously reported to be upregulated by Bcl-xL overexpression in a glioma cell line^14^. Moreover, a dysregulated TGFβ signalling pathway promotes cancer metastasis^31^. To evaluate whether TGFβ may be affected by Bcl-xL-mediated epigenetic modifications and transcription, chromatin immunoprecipitation (ChIP) assay was performed. We found increased H3K4me3 at the promoter region of TGFβ (Fig. 6b) and elevated mRNA levels of TGFβ in BON1-TGL cells overexpressing wild-type Bcl-xL and the two mutants, compared with that in control cells (Fig. 6c).

Next, we analysed TGFβ proteins in the cell lysates and medium from each cell culture with western blot using an antibody that can detect both precursor and mature TGFβ proteins. While both the precursor and mature TGFβ were detected in total cell lysates, the mature TGFβ was mostly observed in the media from BON1-TGL cells overexpressing wild-type Bcl-xL and mutants, but not in the media from the control BON1-TGL cells (Fig. 6d), indicating that TGFβ is produced and secreted from the cells overexpressing Bcl-xL in a Bax/Bak-independent manner.

To examine whether the released TGFβ is responsible for the metastatic function of Bcl-xL, cells pretreated with TGFβ-neutralizing antibodies were subjected to transwell invasion assay in the presence of the TGFβ-neutralizing antibodies. TGFβ-neutralizing antibodies significantly reduced Bcl-xL-mediated invasion, suggesting that secreted TGFβ may be a key factor for metastatic function of Bcl-xL (Fig. 6e). Collectively, these results suggest that Bcl-xL leads to H3K4me3 recruitment to the TGFβ gene promoter, and that activated TGFβ signalling mediates cancer metastasis. Furthermore, transcription inhibition, using actinomycin D (Act-D), blocked the effect of Bcl-xL on invasion, indicating that Bcl-xL-mediated metastatic function requires transcriptional activation (Fig. 6e).

### Nuclear Bcl-xL promotes metastatic features

**Figure 7:**
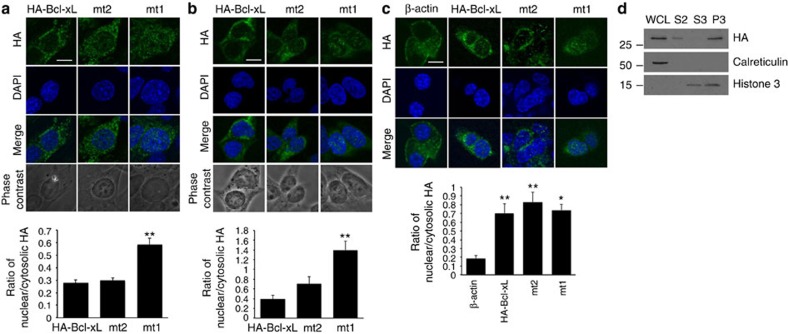
Subcellular localizations of HA-Bcl-xL proteins. (**a**,**b**) wt MEFs (**a**) or BON1-TGL (**b**) cells with HA-Bcl-xL, mt2 or mt1 were cultured on glass coverslips, immunostained with HA and DAPI to identify the nuclei and analysed with confocal microscopy. Bcl-xL was found in multiple subcellular locations. Note the nuclear staining of cells with mt1. Photographs show representative staining from three independent experiments. Scale bar, 10 μm. Original magnification, × 60. The graph shows the ratio of nuclear to cytoplasmic HA fluorescence (background-subtracted) with the s.d. The mean intensities of nuclear HA staining by selecting a region in the nucleus and cytoplasmic HA by subtracting a nuclear region from each cell was determined using Metamorph. This was performed from at least seven pictures taken using confocal microscope. Data were analysed using ANOVA, followed by Tukey's *post hoc* test. ** indicates values significantly different from HA-Bcl-xL at *P*<0.01. (**c**) N134 cells infected with RCASBP-HA-β-actin, HA-Bcl-xL, mt2 or mt1 were cultured on glass coverslips, immunostained with HA (green) and DAPI (blue) to identify the nuclei and analysed using confocal microscopy. Bcl-xL was found in multiple subcellular locations. Scale bar, 10 μm. Original magnification, × 60. The graph shows the ratio of nuclear to cytoplasmic HA fluorescence (background-subtracted) with the s.d. The mean intensities of nuclear HA staining by selecting a region in the nucleus and cytoplasmic HA by subtracting nuclear region from each cell was determined using Metamorph. This was performed from at least seven pictures taken using confocal microscope. Data were analysed using ANOVA, followed by Tukey's *post hoc* test. * indicates values significantly different from β-actin at *P*<0.05. ** indicates values significantly different from β-actin at *P*<0.01. (**d**) N134 cells infected with RCASBP-HA-Bcl-xL were subjected to the biochemical fractionation. Bcl-xL was found in both cytosolic and chromatin/nuclear matrix-enriched fraction. P3, chromatin/nuclear matrix-enriched fraction; S2, cytosolic fraction; S3, soluble nuclear proteins, WCL, whole-cell lysate. Calreticulin, also known as endoplasmic reticulum-resident protein 60, and histone 3 were used as controls for the biochemical fractionation.

Having observed Bcl-xL-induced epigenetic reprograming and transcriptional changes, we next tested whether Bcl-xL may act in the nucleus to regulate metastasis. Besides nuclear localization of Bcl-xL in BON1-TGL liver metastases (Fig. 5c), we also detected some nuclear staining for wild-type Bcl-xL and mutant 2 in MEFs, N134 and BON1-TGL, and prominent nuclear signals of mutant 1 in MEFs and BON1 cells (Fig. 7a–c). In addition to HA staining, Bcl-xL staining of BON1-TGL cells showed the similar nuclear staining of mutant 1 (Supplementary Fig. 4), indicating the presence of intact HA-Bcl-xL in the nucleus. Interestingly, Bcl-xL mutant 1 also exhibited enhanced metastatic ability relative to wild-type Bcl-xL (Fig. 5b). Consistent with the immunofluorescent staining data, biochemical fractionation of N134 cells showed that HA-Bcl-xL was present in both cytosolic fraction and chromatin/nuclear matrix-enriched fraction (Fig. 7d).

**Figure 8:**
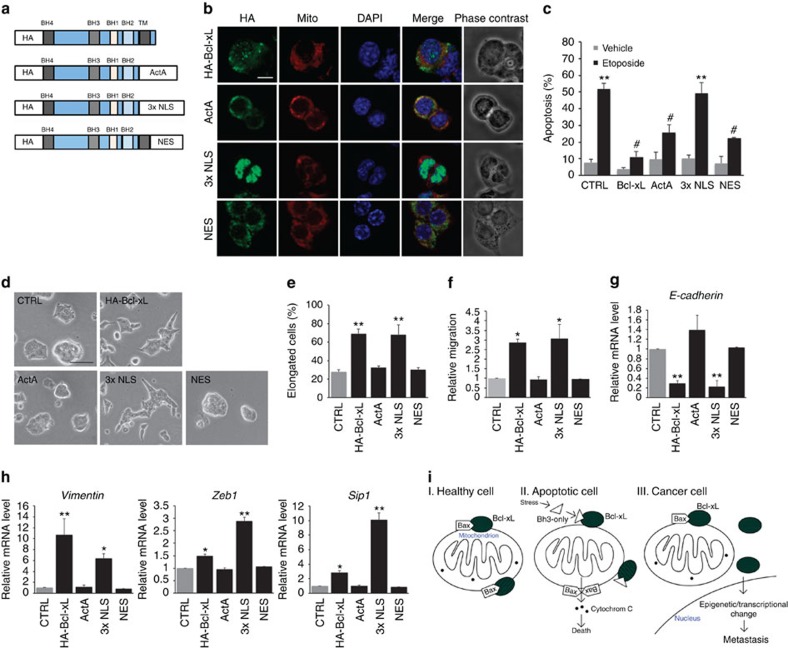
Nuclear Bcl-xL facilitates tumour cell migration and EMT activation. (**a**) Schematic diagram of Bcl-xL wild-type and fusion constructs. Amino-acid sequence for ActA is ILAMLAIGVFSLGAFIKIIQLRKNN, for 3x NLS is 3x (DPKKKRKV) and for NES is LQLPPLERLTLD. BH, Bcl-2 homology domain; TM, transmembrane domain. (**b**) Confocal images of subcellular localization of HA-Bcl-xL in N134 cells following incubation with red-fluorescent Mito Tracker and HA (green) and DAPI (blue) staining. Scale bar, 10 μm. (**c**) Mitochondrial Bcl-xL, not nuclear Bcl-xL, prevents apoptosis. N134 cells were treated with 10-μM etoposide for 24 h and then apoptosis was examined using flow cytometry analysis following Annexin V and PI staining. Data are expressed as the per cent of Annexin V-positive cells relative to that of vehicle-treated cells from three independent experiments (mean±s.e.m., *n*=3). *P<0.01 compared with vehicle-treated CTLR, and #*P*<0.01 compared with etoposide-treated CTRL, one-way ANOVA with Dunnett's *post hoc* analysis. (**d**) Bright-field images of N134 cells. Cells infected with HA-Bcl-xL or 3x NLS changed morphology compared with CTRL cells (HA-β-actin) and cells expressing ActA or NES. Scale bar, 50 μm. (**e**) Quantification of the proportion of elongated cells shown in **d**. Values are means±s.e.m., *N*=4. **P< 0.01 compared with CTRL, one-way ANOVA with Dunnett's *post hoc* test. (**f**) Migration of N134 cells was determined using *in vitro* transwell migration assay, as previously described. Data are expressed as the normalized number of migrated cells relative to that of CTRL cells. Values are mean±s.e.m., *N*=4 independent experiments, *P<0.05 compared with CTRL, ANOVA with Dunnett's *post hoc* test. (**g**,**h**) Quantitative real-time PCR analysis of *E-cadherin* (**g**), *vimentin, zeb1* and *sip1* (**h**) mRNAs in N134 tumour cells infected with RCASBP-*HA-β-actin*, RCASBP-*HA-Bcl-xL*, *HA-Bcl-xL-ActA*, *HA-Bcl-xL*-*3x NLS and HA-Bcl-xL-NES*. Values are mean±s.e.m., *N*=3 independent experiments, *P< 0.05, **P<0.01 compared with CTRL, ANOVA with Dunnett's *post hoc* test. (**i**) Model depicting the roles of Bcl-xL in healthy, apoptotic and cancer cells.

This more prominent nuclear location of Bcl-xL mutant 1 associated with its enhanced metastatic ability relative to wild-type Bcl-xL (Fig. 5b) prompted us to consider whether a nucleus-located Bcl-xL may be responsible for its metastatic function. To test this possibility, we generated N-terminal HA-tagged Bcl-xL fusion proteins by replacing the transmembrane domain (TM) of Bcl-xL with ActA outer mitochondrial membrane-targeting sequence^32^, 3x nuclear localization sequence (3x NLS) or nuclear export sequence (NES; Fig. 8a). Confocal microscopy analysis confirmed that HA-Bcl-xL (wild type) was present in multiple cellular compartments, including the mitochondria and nucleus, in N134 cells, while HA-Bcl-xL-ActA was mostly found colocalized with red fluorescent Mito Tracker in the mitochondria. As expected, HA-Bcl-xL-3x NLS was exclusively localized in the nucleus, and HA-Bcl-xL-NES had normal subcellular localization, except for the nucleus (Fig. 8b). We further confirmed that mitochondrial HA-Bcl-xL-ActA and HA-Bcl-xL-NES prevented N134 tumour cells from apoptosis following etoposide treatment as wild-type Bcl-xL did, and nuclear HA-Bcl-xL-3x NLS failed to do so (Fig. 8c). Wild-type Bcl-xL, mitochondrial HA-Bcl-xL-ActA, nuclear HA-Bcl-xL-3x NLS and HA-Bcl-xL-NES did not significantly change baseline apoptotic rates following vehicle treatment (Fig. 8c). Similar to cells with HA-Bcl-xL (wild type), ∼3 weeks after infection with RCASBP viruses, N134 cells overexpressing nuclear HA-Bcl-xL-3x NLS became elongated. In contrast, cells carrying mitochondria-targeted Bcl-xL or HA-Bcl-xL-NES did not change their morphology (Fig. 8d,e).

Importantly, cells with either wild-type or nucleus-targeted Bcl-xL also possessed increased migration ability, as measured by a transwell migration assay; however, the migration ability of cells with mitochondrial Bcl-xL and HA-Bcl-xL-NES remained similar to control cells (Fig. 8f). In addition to panNET cell lines, we examined whether this nucleus-targeted Bcl-xL promotes cell migration and invasion in two different human breast cancer cell lines, MCF-7 (luminal-like, ER+) and HCC1954 (basal-like, HER2+). Consistent with our findings using N134 cell line, wild-type Bcl-xL and nucleus-targeted Bcl-xL promoted migration and invasion of MCF7 and HCC1954, while mitochondrial Bcl-xL did not (Supplementary Fig. 5a–d). Therefore, these data suggest that Bcl-xL functions in the nucleus to promote migration and invasion of multiple cancer types.

Furthermore, Bcl-xL and nuclear HA-Bcl-xL-3x NLS, but not mitochondrial HA-Bcl-xL-ActA and HA-Bcl-xL-NES, caused EMT-transcriptional changes, such as downregulation of E-cadherin (Fig. 8g) and upregulation of vimentin, zinc-finger E-box-binding homeobox 1 (zeb1) and sip1 (Fig. 8h) mRNA levels in N134 cells. Taken together, these findings strongly suggest that the nuclear pool of Bcl-xL functions specifically to activate EMT and to promote cancer metastasis.

EMT has been reported to confer stemness to epithelial tumour cells^33^,^34^. To determine whether nucleus-targeted Bcl-xL increases stemness, we performed *in vitro* tumour sphere formation assay. Breast cancer cells were selected for this experiment for their common use in this type of assay^35^,^36^. Both wild-type and nucleus-targeted Bcl-xL promoted formation of tumour spheres for MCF-7 and HCC1954 breast cancer cell lines, but mitochondrial Bcl-xL did not (Supplementary Fig. 5e,f). These results suggest that Bcl-xL increases cancer cell stemness characteristics via its activity in the nucleus.

### Bcl-xL expression and localization in human panNET

**Figure 9:**
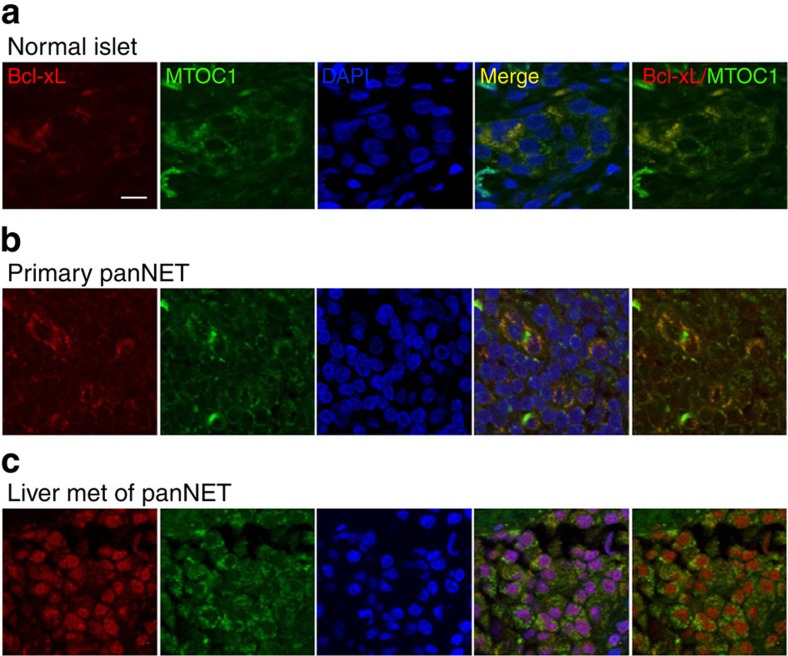
Overexpression and the multiple subcellular localization of Bcl-xL in human panNET. Confocal microscopy images of normal islet (**a**), primary panNET (**b**) and liver metastases of panNET (**c**) stained with Bcl-xL (red), mitochondrial marker MTCO1 (green) and DAPI (blue). Scale bar, 10 μm. Original magnification, × 60.

To evaluate the clinical implication of our findings, we examined the subcellular localization of Bcl-xL in panNET. By immunofluorescent analysis using antibodies against Bcl-xL and MTOC1, a mitochondrial marker, we found that Bcl-xL was overexpressed and colocalized with 4′, 6′-diamidino-2-phenylindole (DAPI), a fluorescent dye that binds strongly to DNA, in three of seven cases of metastatic panNET in the liver (Fig. 9c), while Bcl-xL was mostly colocalized with MTOC1 at the mitochondria in the cytosol of normal pancreatic islets and primary panNETs (Fig. 9a,b). These data demonstrate that Bcl-xL is overexpressed and localized in various cellular compartments in human panNET, and that prominent nuclear Bcl-xL can be found in metastatic panNETs.

## Discussion

Bcl-xL has long been documented for its function in regulating apoptosis both during embryonic development and under pathological conditions^37^. Any role that Bcl-xL might play in tumour metastasis has been ascribed to its anti-apoptotic function; that is, Bcl-xL may increase metastasis by lending survival advantage to the tumour cells during the course of metastasis. We investigated whether the anti-apoptotic function was indeed responsible for Bcl-xL-mediated metastasis. Bcl-xL executes its anti-apoptotic function by binding to and inhibiting the pro-apoptotic activity of Bax/Bak, which are otherwise poised to initiate the apoptotic cell death pathway. To investigate directly the interdependence of the metastatic function and its canonical anti-apoptotic function, we utilized Bax/Bak DKO MEFs and employed specific Bcl-xL mutants (mt1 and mt2) that lost the anti-apoptotic function in this study. *RIP-Tag; RIP-tva* mouse model, which recapitulates multistep tumorigenesis, and various panNET and breast cancer cells, were also used in this study. Our studies reveal that the novel metastatic function of Bcl-xL is not mediated via its canonical anti-apoptotic activity or its ability to suppress anoikis.

Furthermore, we discovered evidence that Bcl-xL promotes metastasis via a nuclear function to exert transcriptional and epigenetic changes (schematic in Fig. 8i). First, mutant 1, which had prominent nuclear localization, possessed the strongest metastatic potential in human BON1-TGL cells in the experimental metastasis assay compared with wild-type and mutant 2 (Fig. 5a,b). The enhanced metastatic effect of mutant 1 may be due to the increased transcriptional activation. Second, there was a 3-week time delay for Bcl-xL to cause EMT in cells. Importantly, wild-type Bcl-xL, mutant 1 and mutant 2 increased global levels of H3K4me3 (Fig. 6a), which is known to be a mark for transcriptional activation. Third, mutant 1 significantly recruited H3K4me3 to the TGFβ promoter (Fig. 6b). Bcl-xL overexpression caused upregulation of TGFβ in BON1-TGL tumour cells in a Bax/Bak-independent manner (Fig. 6c,d), and both TGFβ-neutralizing antibodies and Actinomycin D blocked Bcl-xL-mediated invasion (Fig. 6e). The release of TGFβ may contribute to metastasis in an autocrine and/or paracrine manner by leading to EMT of tumour cells, inhibiting cell adhesion, inducing immunosuppression and angiogenesis and promoting the degradation of the extracellular matrix. Fourth, nuclear Bcl-xL, but not mitochondrial Bcl-xL and cytosolic Bcl-xL, promoted EMT and migration of panNET and breast cancer cells, although nuclear Bcl-xL failed to protect cells from apoptosis (Fig. 8c–h). Lastly, nuclear Bcl-xL, but not mitochondrial Bcl-xL, increased the stemness of breast cancer cells (Supplementary Fig. 5e,f). Altogether, these findings indicate that the metastatic function Bcl-xL is independent of its canonical anti-apoptotic activity and requires a novel nuclear function.

The evidence presented here demonstrates that Bcl-xL promotes metastasis independently from its canonical anti-apoptotic function and its mitochondria localization in panNET, breast cancer cell lines and mouse models. In accordance with this finding, we detected nuclear staining of Bcl-xL in metastatic human panNET samples (Fig. 9), consistent with a previous report, which showed that nuclear Bcl-xL in human non-small cell carcinomas correlates with distant metastasis^38^. Since Bcl-xL is overexpressed in a variety of cancers^39^,^40^,^41^,^42^,^43^,^44^,^45^, Bcl-xL may promote metastasis via nuclear activity in human tumours of other tissue types as well.

Our finding of apoptosis-independent functions of Bcl-xL has important clinical implications, as it may help explain why clinical trials using drugs to block anti-apoptotic roles of Bcl-xL has not been effective in halting cancer progression. For example, the small-molecule inhibitor ABT-737 is a BH3 mimetic that can block Bcl-xL (and Bcl-2 and Bcl-w) from binding to Bax/Bak (refs 5, 8, 46); however, clinical trials using ABT-263, an orally bioavailable derivative of ABT-737, has led to poor response rates^47^,^48^. We found that ABT-737 could not block the cell migration induced by Bcl-xL (Fig. 2g), and that binding to Bax/Bak was dispensible for the metastatic function of Bcl-xL. Therefore, small molecules that inhibit both mitochondrial and nuclear functions of Bcl-xL may have a more promising translational impact for cancer therapeutics than the current inhibitors of Bcl-xL.

## Methods

### Cell culture

Generation of wild-type MEFs, Bax/Bak DKO MEFs^49^,^50^ and N134 cell line^12^ has been described. The human panNET cancer cell line, BON1, was provided by Chris Harris^28^,^51^. The BON1 cell line was infected with virus carrying TGL, which was provided by Inna Serganova and Ronald Blasberg^29^. GFP-positive cells have been sorted using BD Biosciences FACS-DiVa Cell Sorter to generate pure BON1-TGL cells. MEFs, N134, BON1-TGL, MCF-7 and HCC1954 were cultured in DMEM supplemented with 10% fetal bovine serum (FBS), 0.2 mM L-glutamine and 1% penicillin/streptomycin. Cells were tested negative for mycoplasma contamination.

### Animal experiments

Generation of *RIP-Tag; RIP-tva* mice has been described^12^. NSG mice were generated by the Jackson Laboratory. All mice were housed in accordance with the institutional guidelines. All procedures involving mice were approved by the institutional animal care and use committee of Weill Cornell Medicine. For the experimental metastasis assay, either 0.5 × 10^5^ MEF cells in 400 μl PBS were injected into the tail veins of male NSG mice at the age of 7–8 weeks (*n*=7 CTRL group, *n*=8 Bcl-xL group) or 1 × 10^6^ BON1-TGL cells in 100 μl PBS were injected into the left ventricle of male NSG mice at the age of 7–8 weeks (*n*=5 each group), as described^52^. Mice were subjected to *in vivo* bioluminescent imaging using the *In Vivo* Imaging System Spectrum (PerkinElmer) at 0, 1, 2, 3, 5 days and 1, 2, 3, 4 weeks after tumour cells were injected, as described previously^53^.

### Cloning of RCAS and retroviral vectors

RCASBP is a replication-competent avian leucosis virus with a splice acceptor and the Bryan-RSV pol gene. RCASBP-Y-DEST has been described^54^. pENTR3C-*HA-Bcl-xL* was generated and provided by Matthew Van Brocklin (unpublished). Bcl-xL mutants were generated using the QuickChange Lightning Site-Directed Mutagenesis kit (Agilent Technology) and confirmed by sequencing. pENTR3C-*HA-Bcl-xL*, *mt1* and *mt2* were recombined either with RCASY-DEST or pQCXIP-puro-DEST, using LR Clonase (Invitrogen) to generate RCASBP-*HA-Bcl-xL, mt1* and *mt2* or pQCXIP-puro-*HA-Bcl-xL, mt1* and *mt2*, respectively. Inserted regions were confirmed using DNA sequencing. To generate *Bcl-xL-ΔTM, Bcl-xL-ΔTM-ActA, Bcl-xL-ΔTM-3x NLS*, NotI and AscI, restriction enzyme sites were added right before the stop codon of pENTR3C-*HA-Bcl-xL* by using the QuickChange Lightning Site-Directed Mutagenesis kit (Agilent Technology). The transmembrane domain was further deleted by using the QuickChange Lightning Site-Directed Mutagenesis kit. PCR-generated ActA and 3x NLS (DNA templates provided by David Andrews^32^ and Invitrogen, respectively) were cloned into pENTR3C-*HA-Bcl-xL-ΔTM* using NotI and AscI sites. For *Bcl-xL-NES*, pENTR3C-*HA-Bcl-xL-NES* was synthesized and verified by GENEWIZ Inc. Constructs were confirmed by DNA sequencing and then were recombined into RCASBP-Y-DEST using LR Clonase (Invitrogen) according to the manufacturer's instruction.

### Expression vectors and cell transfection/infection

Vial propagation, titre determination and infection were previously described^12^. To generate retroviruses, the empty vector (as control) and other pQCXIP-based plasmids were transfected to the GPG29 amphotropic packaging cell line^55^ by using Lipofectamine 2000 (Invitrogen). Viruses were harvested at 48, 72 and 96 h after transfection and the filtered viral supernatant was used to infect cells in the presence of 8 μg ml^−1^ polybrene. Infected cells were selected with 2 or 0.5 μg ml^−1^ puromycin for MEFs or BON1-TGL cells, respectively.

### Tissue preparation and immunohistochemical analysis

Mouse tissues were removed and fixed in 10% buffered formalin overnight at room temperature. Fixed tissues were cut into 5-μm sections at Histoserv Inc. Formalin-fixed/paraffin-embedded sections were deparaffinized and rehydrated by passage through a graded xylene/ethanol series before staining. Immunochemistry was examined using theVECTASTAIN Elite ABC kit following the manufacturer's instructions. The primary antibodies used were rabbit anti-synaptophysin (1:100; Vector Labs, VP-S284 or 1:100; Neomarker, RM-9111), HA (1:400, Cell Signaling Technology, 3724), Bcl-xL (1:500, Cell Signaling Technology, 2764) and luciferase (1:100, Santa Cruz Biotechnology, 251–550).

### Immunofluorescent analysis

Cells (MEFs, N134 and BON1-TGL) were cultured on glass coverslips for 24 h before fixation in 4% paraformaldehyde in PBS for 10 min. After permeabilizing and blocking in 0.5% BSA with PBS plus 0.025 % Triton X-100, 0.02% NaN3 and 0.3 μM DAPI for 30 min, coverslips were incubated with primary antibodies overnight at 4 °C. The coverslips were then washed three times with PBS for 15 min, followed by incubation with the secondary antibodies coupled to different fluorophores at room temperature in the dark for 1 h, and were washed three times with PBS for 15 min. Coverslips were mounted with Vectashield mounting medium (Vector Labs) and were examined using BX51 fluorescence microscopy (Olympus) or confocal microscopy (Leica). The primary antibodies were anti-HA (1:500; Cell Signaling Technology, 2367). For mitotracker staining, cells were incubated with 500 nM of MitoTracker Red CMXRos (Invitrogen, M-7512) at 37 °C for 15 min before fixation.

Retrospective and prospective review of well-differentiated neuroendocrine tumours of the pancreas (G3) was performed using the pathology files and pancreatic cancer database at the authors' institutions with institutional review board (IRB) approval. For human panNET tissue section staining, formalin-fixed/paraffin-embedded sections were deparaffinized and rehydrated by passage through a graded xylene/ethanol series and blocked in 0.5% BSA with PBS plus 0.025% Triton X-100, 0.02% NaN3 and 0.3 μM DAPI for 30 min. The sections were incubated with primary antibodies for anti-HA (1:200; Cell Signaling Technology, 2367) and anti-MTCO1 (1:200; Abcam, ab14705) overnight at 4 °C, washed three times with PBS for 15 min, incubated with the secondary antibodies coupled to different fluorophores at room temperature in the dark for 1 h, washed three times with PBS for 15 min, mounted with Vectashield mounting medium (Vector Labs) and examined using confocal microscopy (Leica).

### Flow cytometry and apoptosis assay

Apoptosis was measured with annexin V and propidium iodide staining using the Alexa Fluor 488 annexin V/Dead Cell Apoptosis Kit (Invitrogen) according to the manufacturer's instructions. MEFs were treated with either 50 μM of etoposide or 60 J m^−2^ of UV (254 nm) for 24 h to induce apoptosis. N134 cells were incubated with 10 μM of etoposide for 24 h, and BON1-TGL cells were treated with 300 J m^−2^ of UV (254 nm). Stained cells with Alexa Fluor 488 annexin V/Dead Cell Apoptosis Kit were analysed with flow cytometry using a BD LSR II flow Cytometer. Annexin V or propidium iodide-positive cells were counted as apoptotic or dead cells.

### Western blot analysis and immunoprecipitation

Cells (MEFs, N134 and BON1-TGL) were lysed in NP-40 buffer (100 mM NaCl, 100 mM Tris (pH 8.2) and 0.5% NP-40) supplemented with a protease inhibitor mixture and PhosSTOP (Roche). Proteins were quantified with Bradford assay (Bio-Rad). Equal amounts of proteins were separated with SDS–PAGE and transferred to nitrocellulose membranes. To visualize equal protein loading, blots were stained with Ponceau S. Blots were incubated in 5% non-fat milk in TBST, probed with primary antibodies to E-cadherin (1:1,000, BD, 610181), α-tubulin (1:1,000, Sigma, T5168), HA (1:1,000, Cell Signaling Technology, 2367), Bax (1:1,000, Cell Signaling Technology, 2772), Bcl-xL (1:1,000, Cell Signaling Technology, 2764) and TGFβ (1:1,000, Cell Signaling Technology, 3711), and then were incubated with horseradish peroxidase-conjugated secondary antibodies. Protein bands were visualized by enhanced chemical luminescence (Pierce). For western blot analysis of H3K4me3 (1 μg ml^−1^, Abcam, ab8580) and total histone 3 (1:1,000, Millipore, 07–690), histones were obtained by acid extraction according to the Abcam's instruction (http://www.abcam.com/protocols/histone-extraction-protocol-for-western-blot). For Bax immunoprecipitation, 500 μg of total protein from cell lysates was incubated with the anti-Bax antibody (Cell Signaling Technology, 2772) overnight on a rocking platform at 4 °C and then protein A/G Sepharose beads (Santa Cruz Biotechnology, SC-2003) were added for 2 h at 4 °C. Immunoprecipitates were subjected to western blot analysis and probed with anti-HA antibody (1:1,000, Cell Signaling Technology, 2367) and anti-Bax antibody. For HA immunoprecipitation, 1,800 μg of total protein from cell lysates was incubated with anti-HA magnetic beads (Pierce) for 1 h on a rocking platform at 4 °C. Immunoprecipitates were subjected to western blot analysis and probed with anti-Bax antibody (Cell Signaling Technology, 2772). Full western blots are presented in Supplementary Fig. 6.

### *In vitro* transwell migration and invasion assay

For the migration assay, 5 × 10^4^ of cells (MEFs, MCF7 or HCC1954) were seeded in the upper chambers of 8-μm porous polycarbonate membranes (Corning, 3422) with DMEM containing 2% FBS, 0.2 mM L-glutamine and 1% penicillin/streptomycin. The lower chambers were filled with DMEM containing 10% FBS, 0.2 mM L-glutamine and 1% penicillin/streptomycin. After 4 h (for MEFs), 36 h (for MCF7) or 8 h (for HCC1954) of incubation, cells migrating to the opposite side of the upper chambers were fixed, stained with 0.1% crystal violet for 30 min and counted in eight fields under × 20 magnification. For N134, 0.5 × 10^6^ or 1 × 10^6^ of cells were seeded in the upper chambers of 8-μm porous polycarbonate membranes with DMEM containing 2% FBS, 0.2 mM L-glutamine and 1% penicillin/streptomycin. The lower chambers were filled with DMEM containing 10% FBS, 0.2 mM L-glutamine and 1% penicillin/streptomycin. After 72 h of incubation, cells migrating to the bottom chambers were fixed, stained with 0.1% crystal violet for 30 min and counted in eight fields under × 10 magnification. For the invasion assay, 2 × 10^4^ BON1 cells were seeded in the matrigel-coated transwell chambers (BD Biosciences) with DMEM containing 1% FBS, 0.2 mM L-glutamine and 1% penicillin/streptomycin. The lower chambers were filled with DMEM containing 10% FBS, 0.2 mM L-glutamine and 1% penicillin/streptomycin. After 24 h of incubation, cells on the opposite side of the chambers were fixed, stained with 0.1% crystal violet for 30 min and counted in eight fields under × 20 magnification. For breast cancer cell invasion assay, 5 × 10^4^ breast cancer cells were seeded to the upper chambers, which were pre-coated with 20% matrigel. MCF7 and HCC1954 cells on the bottom chambers were counted after 48 and 24 h, respectively.

### Tumour sphere formation assay

Overall, 1 × 10^4^ cells (MCF7 and HCC1954) were plated in ultralow attachment six-well plates with tumour-sphere media containing DMEM/F12, supplemented with 2 mM L-glutamine, 100 U ml^−1^ penicillin, 100 U ml^−1^ streptomycin, 20 ng ml^−1^ recombinant human epidermal growth factor (EGF; R&D Systems), 10 ng ml^−1^ recombinant human basic fibroblast growth factor (bFGF; R&D Systems or Novoprotein) and 1x B27 supplement (BD) for 14 days.

### Quantitative real-time reverse transcription PCR

mRNA was isolated from cells (N134 and BON1-TGL) grown on 6- or 10-cm plates using Trizol with DNase I treatment (Invitrogen) or RNeasy mini kit (Qiagen) containing gDNA Eliminator spin columns. cDNA was generated using the SuperScript III First-strand synthesis system with random hexamers (Invitrogen), and power SYBR green (Invitrogen)-based quantitative real-time PCR was performed using primer specific for E-cadherin (mouse, forward: 5′CAGGTCTCCTCATGGCTTTGC-3′, reverse: 5′-CTTCCGAAAAGAAGGCTGTCC-3′), Sip1 (mouse, forward: 5′-ATGGCAACACATGGGTTTAGTGGC-3′, reverse: 5′-ATTGGACTCTGAGCAGATGGGTGT-3′), β-actin (mouse, forward: 5′-ATAGGAGTCCTTCTGACCCATTCC-3′, reverse: 5′-ATGACGATATCGCTGCGCTGGT-3′), β2M (mouse, forward: 5′-ATGCTGAAGAACGGGAAAAA-3′, reverse: 5′-CAGTCTCAGTGGGGGTGAAT-3′) or S16 (mouse, forward: 5′-AGGAGCGATTTGCTGGTGTGGA-3′, reverse: 5′-GCTACCAGGCCTTTGAGATGGA-3′) with the comparative *C*_T_ method (ΔΔC_T_; ABI).

### Subcellular fractionation

Subcellular fractionation was performed as previously described^56^ with modifications. N134 cells were resuspended in buffer A (10 mM HEPES (pH 7.9), 10 mM KCl, 1.5 mM MgCl_2_, 0.34 M sucrose, 10% glycerol, 1 mM dithiothreitol and a protease inhibitor mixture) and incubated for 5 min on ice following 0.1% Triton X-100 addition. Nuclei were isolated in the pellet by centrifugation (4 min, 1,300*g*, 4 °C) and the supernatant was further cleared by centrifugation (15 min, 20,000*g*, 4 °C) to obtain soluble cytosolic fraction (S2). Nuclei were washed with buffer A and then resuspended in buffer B (3 mM EDTA, 0.2 mM EGTA, 1 mM dithiothreitol and a protease inhibitor mixture) for 30 min on ice.

The pellet enriched with chromatin and nuclear matrix was isolated with centrifugation (4 min, 1,700*g*, 4 °C) and the supernatant fraction (S3) contained soluble nuclear proteins. After washing the pellet with buffer B, cleared P3 was collected using centrifugation (4 min, 1,700*g*, 4 °C). The final chromatin pellet fraction (P3) was resuspended in Laemmli buffer and sonicated for 15 s twice.

### Chromatin immunoprecipitation

ChIP assays were performed as described^57^ with modifications. For each immunoprecipitation, BON1 cells from four dishes were combined. Cells were sonicated using a Sonifier (Branson) for 10 min (alternating cycles of 15 s of sonication and 45 s of rest). Chromatin was immunoprecipitated with 2 μg of a rabbit polyclonal anti-H3K4me3 antibody (Abcam, ab8580) or normal rabbit IgG (Vector Lab). Quantitative real-time PCR was performed using primer specific for TGFβ promoter regions (forward: 5′-ACCAGAGAAAGAGGACCAGG-3′, reverse: 5′-TTGTTTCCCAGCCTGACTCT-3′).

### Statistical analysis

The numbers of mice used for *in vivo* experiments were determined on the basis of our pilot experiments and previous experience with similar types of experiments. Mice were randomized into control and treatment groups using a random number table. All *in vitro* experiments were repeated at least three times, unless indicated otherwise. Investigators who counted the lymph node metastasis were blinded with respect to the treatment allocation. The normality of numerical data was tested with D'Agostino-Pearson test. Differences between two groups were compared by paired Student's *t*-test or Wilcoxon rank-sum test, as appropriate. Differences among multiple groups were compared using one-way ANOVA, followed by either Dunnett's, Tukey's or Holm's *post hoc* test. The association between categorical data was tested using Fisher's exact test. All analyses were performed in GraphPad Prism or SAS9.4.

## Additional information

**How to cite this article:** Choi, S. *et al*. Bcl-xL promotes metastasis independent of its anti-apoptotic activity. *Nat. Commun.* 7:10384 doi: 10.1038/ncomms10384 (2016).

## Supplementary Material

Supplementary Information Supplementary Figures 1-6.

## Figures and Tables

**Table 1:** Impact of Bcl-xL overexpression on experimental metastasis *in vivo*.

Bax/Bak DKO MEFs	Lung metastases	Tumour foci (per mouse)
Vector	3/7 Mice (43%)	2.3±1.2
Bcl-xL	6/8 Mice (75%)	16.3±9.4*

DKO, double knockout; MEFs, mouse embryonic fibroblasts.

Bax/Bak DKO MEFs overexpressing Bcl-xL developed more micrometastatic foci compared with control Bax/Bak DKO MEFs (*Wilcoxon rank sum test, *P* value=0.0046).
