# Supplementary material for: Bcl-xL promotes metastasis independent of its anti-apoptotic activity
Source: Nat Commun. 2016 Jan 20;7:10384. doi: 10.1038/ncomms10384 (PMC4735924; doi:10.1038/ncomms10384)
Supplement: Supplementary Information — Supplementary Figures 1-6. [file ncomms10384-s1.pdf]

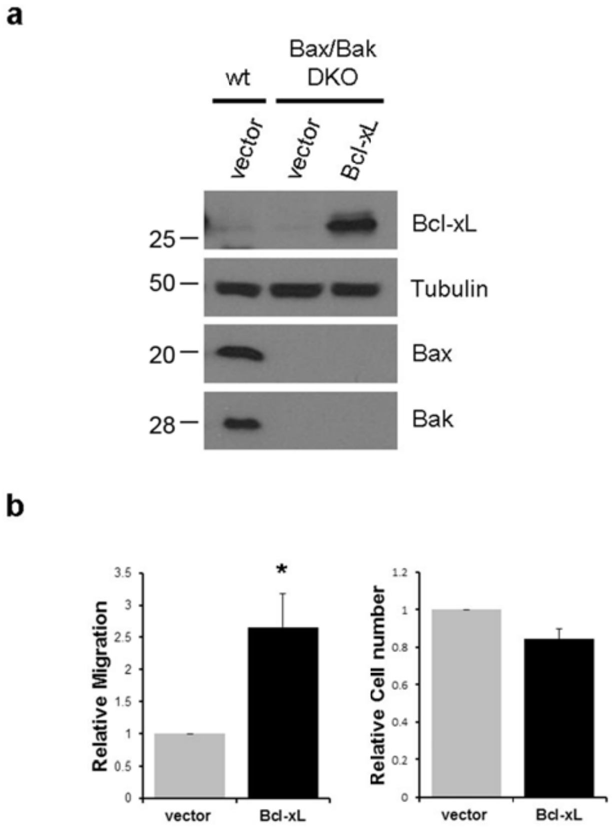

Supplementary Figure 1. Bcl-xL promotes migration in the absence of pro-apoptotic proteins Bax/Bak in MEFs

(a) Western blot analysis for the level of Bcl-xL proteins in wt MEFs and Bax/Bak DKO MEFs overexpressing control vector (pQCXIP) or Bcl-xL. α-tubulin was used a loading control. (b) Migration of Bax/Bak DKO MEFs overexpressing control vector or Bcl-xL was determined using *in vitro* transwell migration chamber with a serum gradient (2% to 10%). 5 x 10^4 cells were seeded in the upper chambers of the transwell inserts. Four hours later, cells attached on the top of the upper chambers were removed. Following crystal violets staining, cells on the bottom surface of the transwell inserts were counted from 8 randomly picked fields in four independent experiments. Error bars represent s.e.m. * P=0.04 relative to control (vector), two-sided t test.

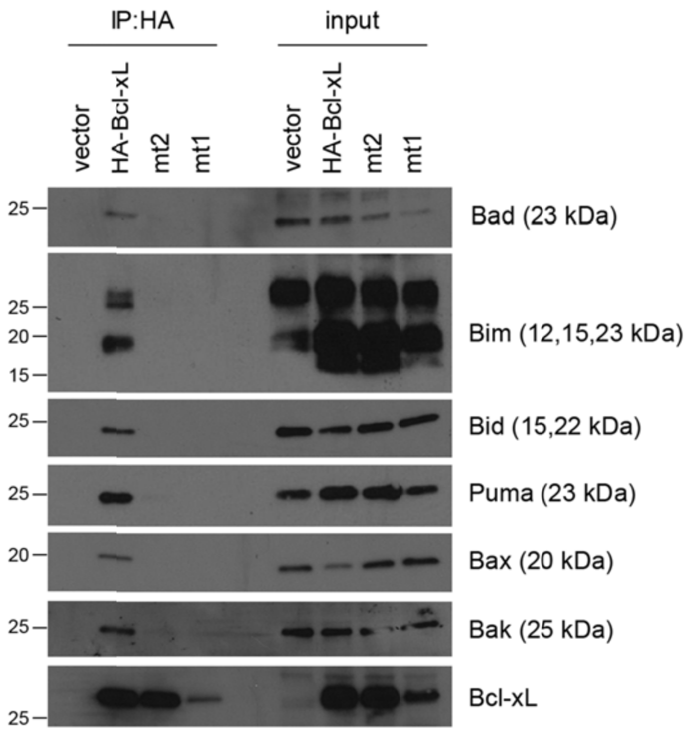

Supplementary Figure 2. Lack of binding ability of Bcl-xL mutants to pro-apoptotic Bcl-2 family proteins

Cell lysates of BON1-TGL overexpressing control vector, HA-Bcl-xL (wt), HA-Bcl-xL mt2, and HA-Bcl-xL mt1 were subjected to immunoprecipitation with HA antibody and then followed by Western blot analysis to detect six pro-apoptotic Bcl-2 family proteins; Bad, Bim, Bid, Puma, Bax, and Bak. Both Bcl-xL mutants (mt2 and mt1) lost the ability to bind these six pro-apoptotic Bcl-2 family members. Data shown are representative of two independent experiments.

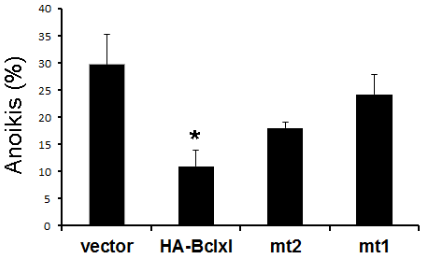

Supplementary Figure 3. Bcl-xL mutants defective in the anti-apoptotic function fail to protect anoikis

wt MEFs overexpressing the control vector, HA-Bcl-xL, HA-Bcl-xL-mt2, and HA-Bcl-xL-mt1 were cultured for 3 days in ultra-low attachment plates and then apoptosis was examined by flow cytometry analysis following Annexin V and PI staining. Only HA-Bcl-xL (wild type) prevented anoikis. * $P<0.05$ relative to the vector CTRL. N =4 independent experiments, and error bar represents s.e.m. Data were analyzed using ANOVA, followed by Dunnett’s post hoc test.

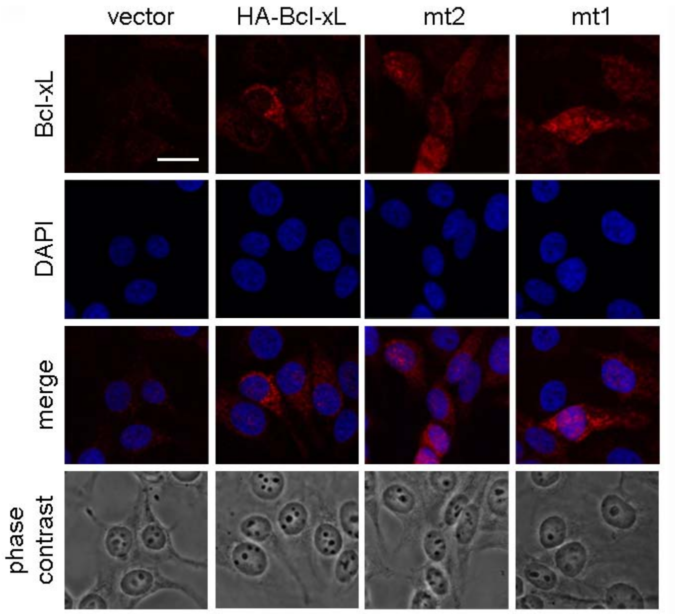

Supplementary Figure 4. Various subcellular localizations of HA-Bcl-xL, mt2, and mt1 in BON1-TGL

BON1-TGL cells with vector alone, HA-Bcl-xL, mt2, or mt1 were cultured on glass cover slips, immunostained with Bcl-xL (red) and DAPI (blue) to identify the nuclei, and analyzed by confocal microscopy. Bcl-xL was found in multiple subcellular locations. Photographs show representative staining from three independent experiments. Scale bar, 10μm. Original magnification, 60X.

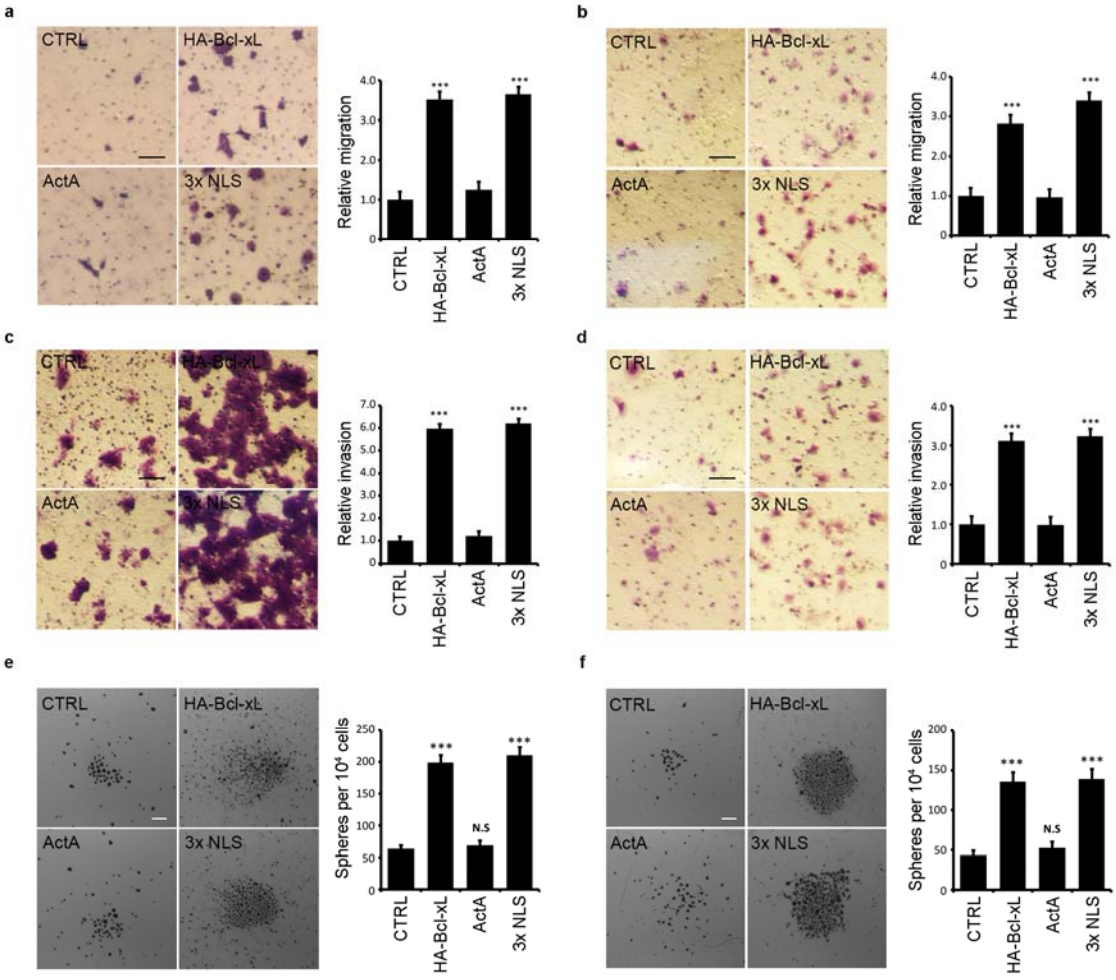

Supplementary Figure 5. Nuclear Bcl-xL promotes migration and invasion and increases tumor spheres formation of breast cancer cell lines

(a and b) Migration of MCF-7 (a) and HCC1954 (b) breast cancer cells infected with CTRL (vector alone), HA-Bcl-xL, HA-Bcl-xL-ActA, and HA-Bcl-xL-3x NLS was determined using *in vitro* transwell migration assay. 0.5 x10^5 cells were seeded to the upper chambers. Data were expressed as the normalized number of migrated cells in the bottom chambers in eight fields under after 36h (MCF7) and 8h (HCC1954) relative to that of control cells (CTRL). *** indicates values significantly different from CTRL at P<0.001, Student's t-test. Values are means ± s.e.m., N =5. Scale bar, 100μm. (c and d)

Invasion of MCF-7 (c) and HCC1954 (d) breast cancer cells infected with CTRL, HA-Bcl-xL, HA-Bcl-xL-ActA, and HA-Bcl-xL-3x NLS was determined using *in vitro* transwell invasion assay. 0.5 $x10^5$ cells were seeded to the upper chambers, which were pre-coated with 20% matrigel. Data were expressed as the normalized number of migrated cells in the bottom chambers in eight fields under after 48h (MCF7) and 24h (HCC1954) relative to that of vector cells. *** indicates values significantly different from CTRL at P<0.001, Student's t-test. Values are means ± s.e.m., N =5. Scale bar, 100μm. (e and f) $1x10^4$ of MCF7 (e) and HCC1954 (f) cells were cultured for 14 days in ultra-low attachment 6-well plates with tumor-sphere media. The numbers of sphere greater than 50 μm in diameter were counted under a microscope. *** indicates values significantly different from CTRL at P<0.001, Student's t-test. Values are means ± s.e.m., N =3. Scale bar, 1000μm.

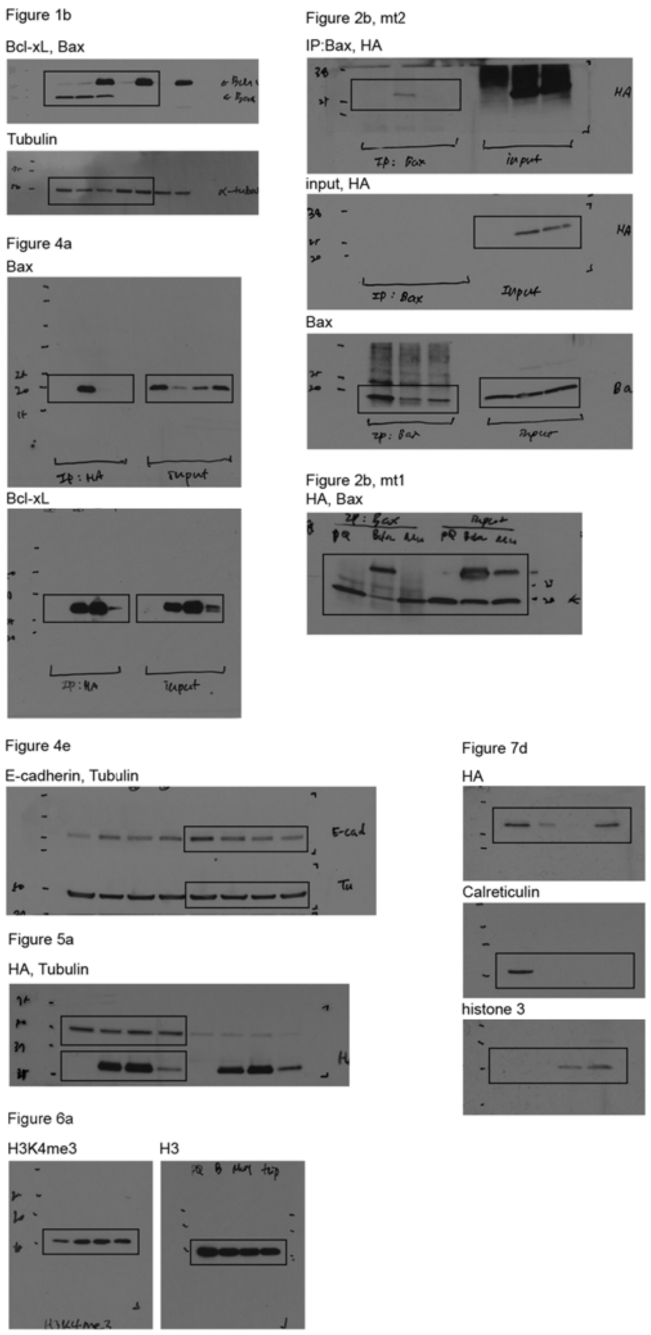

Supplementary Figure 6. Original full scans of Western blots related to respective figures as indicated.
